# Titania nanoparticles for photocatalytic degradation of ethanol under simulated solar light

**DOI:** 10.3762/bjnano.14.51

**Published:** 2023-05-22

**Authors:** Evghenii Goncearenco, Iuliana P Morjan, Claudiu Teodor Fleaca, Florian Dumitrache, Elena Dutu, Monica Scarisoreanu, Valentin Serban Teodorescu, Alexandra Sandulescu, Crina Anastasescu, Ioan Balint

**Affiliations:** 1 National Institute for Lasers, Plasma and Radiation Physics, Atomistilor Str., No. 409, 077125, Bucharest-Magurele, Romania; 2 National Institute of Materials Physics, Atomistilor Str., No. 405A, 077125, Bucharest-Magurele, Romania; 3 Romanian Academy, Inst. Phys. Chem. Ilie Murgulescu, 202 Spl. Independentei, 060021, Bucharest, Romania

**Keywords:** ethanol, H_2_ production, laser pyrolysis, photocatalyst, TiO_2_ nanoparticles

## Abstract

TiO_2_ nanoparticles were synthesized by laser pyrolysis from TiCl_4_ vapor in air in the presence of ethylene as sensitizer at different working pressures (250–850 mbar) with and without further calcination at 450 °C. The obtained powders were analyzed by energy-dispersive X-ray spectroscopy, X-ray photoelectron spectroscopy, X-ray diffractometry, and transmission electron microscopy. Also, specific surface area and photoluminescence with optical absorbance were evaluated. By varying the synthesis parameters (especially the working pressure), different TiO_2_ nanopowders were obtained, whose photodegradation properties were tested compared to a commercial Degussa P25 sample. Two series of samples were obtained. Series “a” includes thermally treated TiO_2_ nanoparticles (to remove impurities) that have different proportions of the anatase phase (41.12–90.74%) mixed with rutile and small crystallite sizes of 11–22 nm. Series “b” series represents nanoparticles with high purity, which did not require thermal treatment after synthesis (ca. 1 atom % of impurities). These nanoparticles show an increased anatase phase content (77.33–87.42%) and crystallite sizes of 23–45 nm. The TEM images showed that in both series small crystallites form spheroidal nanoparticles with dimensions of 40–80 nm, whose number increases with increasing the working pressure. The photocatalytic properties have been investigated regarding the photodegradation of ethanol vapors in Ar with 0.3% O_2_ using P25 powder as reference under simulated solar light. During the irradiation H_2_ gas production has been detected for the samples from series “b”, whereas the CO_2_ evolution was observed for all samples from series “a”.

## Introduction

Semiconductor materials are widely used, from electronic components to substances that catalyze decomposition processes. They have a bandgap that varies from one material to another. Titanium dioxide is a semiconductor material and has been investigated, at first, for solar cells [1–4] and as optoelectronic component [5–7]. In recent years, it has been found that TiO_2_ shows a high photocatalytic efficiency in the decomposition of pollutant substances such as dye wastewater [8–11], soiling [12], and harmful organic materials [13–15]. Also, TiO_2_ powders show promising results for the decomposition of ethanol in various environments [16–18]. Moreover, investigations have shown the possibility for applying TiO_2_ in hydrogen production by water decomposition [19–23]. Given the TiO_2_ bandgap, it is considered a low-efficiency material in photodriven water splitting, because only 3% of the solar light can be used. Different approaches were tried to reduce the bandgap [24] by doping with, for example, nitrogen [17].

Recent investigations have shown a possible application of TiO_2_ for the photocatalytic production of hydrogen from water with the aid of sacrificial agents, such as methanol, ethanol, or glycols [21–22]. There are many studies carried out in gas and liquid phases concerning the photodegradation of ethanol through TiO_2_-based materials, targeting both hydrogen production [25–26] and the photocatalytic oxidation of ethanol to CO_2_ [27–28]. Hydrogen production and depollution via ethanol photodegradation are of great interest because ethanol is an inexpensive compound and can be produced by biomass. However, it can be also largely found as pollutant in air and wastewater emerging from industrial activities.

There are several pathways to convert ethanol to hydrogen, namely thermochemical, hydrothermal electrochemical, and photochemical methods [25]. Ethanol obtained from biomass is a renewable resource, and hydrogen has a high energy content and does not produce greenhouse gases by burning. Hence, it is an ideal combustible for the future [25].

TiO_2_ has some advantages over commonly used catalytic Pt- or Pt-doped materials. It is inexpensive, non-toxic, stable in different solvents and under irradiation, and it can be doped with different elements according to specific necessities. TiO_2_ can crystalize in three different crystallographic structures, namely anatase, rutile, and brookite [29–30]. The differences in crystal structure are reflected in direct or indirect electron transitions. The bandgaps of anatase and rutile differ only by 0.2 eV, but can influence significantly the creation of electron–hole pairs, resulting in an increase of the photocatalytic activity. Anatase has a higher decomposition efficiency than rutile [14,31], while the highest photocatalytic activity has been found in mixed anatase/rutile TiO_2_ [13,32]. One possible explanation is that the difference in the crystal structure and chemical bonding results in different ionization potentials and electron affinities. Exploiting these differences could promote the fabrication of new devices with higher efficiency in electron–hole separation [33].

There are a lot of methods to obtain TiO_2_ powder, from chemical reactions in solvents [34–36] to simple oxidation at high temperatures [37]. Every method has its particular yield and productivity, which are in some cases extremely low compared to a continuous flow method such as laser pyrolysis which, in the case of the studied powders, allows for a productivity of 1 g/h with the possibility of upscaling to an industrial level by increasing the reaction area. Another point is to find the ratio between anatase and rutile that yields the highest photocatalytic activity. Thus, the main scope of this study is to find the best process parameters for the pyrolysis synthesis of TiO_2_ powders. Another part is to obtain powders with specific mixtures of the crystallographic phases (anatase/rutile) that yield the highest photocatalytic decomposition of ethanol as harmful compound in gaseous or liquid media, that is air and wastewater.

## Results and Discussion

### Powder characterization

The main chemical reaction of the TiCl_4_ precursor in laser pyrolysis in the presence of synthetic air can be described as:


[1] $${\text{TiCl}}_{4}+{\text{O}}_{2}\to {\text{TiO}}_{2}+2{\text{Cl}}_{2}.$$

The raw TiO_2_ powders contain some carbon (from the decomposition of the ethylene sensitizer) and chlorine impurities, whose amount it is greatly diminished by calcination in air at 450 °C for 5 h. To certify this, a composition investigation by energy-dispersive X-ray spectroscopy (EDS) has been done. Calcined TiO_2_ powders contain titanium and oxygen and small traces of impurities (ca. 1%). The compositions of all calcined powder samples are presented in Table 1. Theoretically, the ratio between O and Ti should be 2:1. However, because of impurities and crystal structure imperfections (point defects), there are some deviations. The small oxygen deficiency observed even after calcination is related to remaining chlorine impurities and Ti^3+^ ions that resisted calcination.

**Table 1:** Composition of the obtained TiO_2_ powders.

Sample	Ti [atom %]	O [atom %]	Impurities [atom %]

TO-250-a	33.91	65.06	1.03
TO-450-a	33.72	65.14	1.14
TO-650-a	33.21	65.39	1.40
TO-850-a	33.91	65.31	0.78

TO-250-b	34.50	64.21	1.29
TO-450-b	32.49	67.01	0.50
TO-650-b	33.75	65.60	0.65
TO-850-b	33.01	65.71	1.28

**Figure 1:**
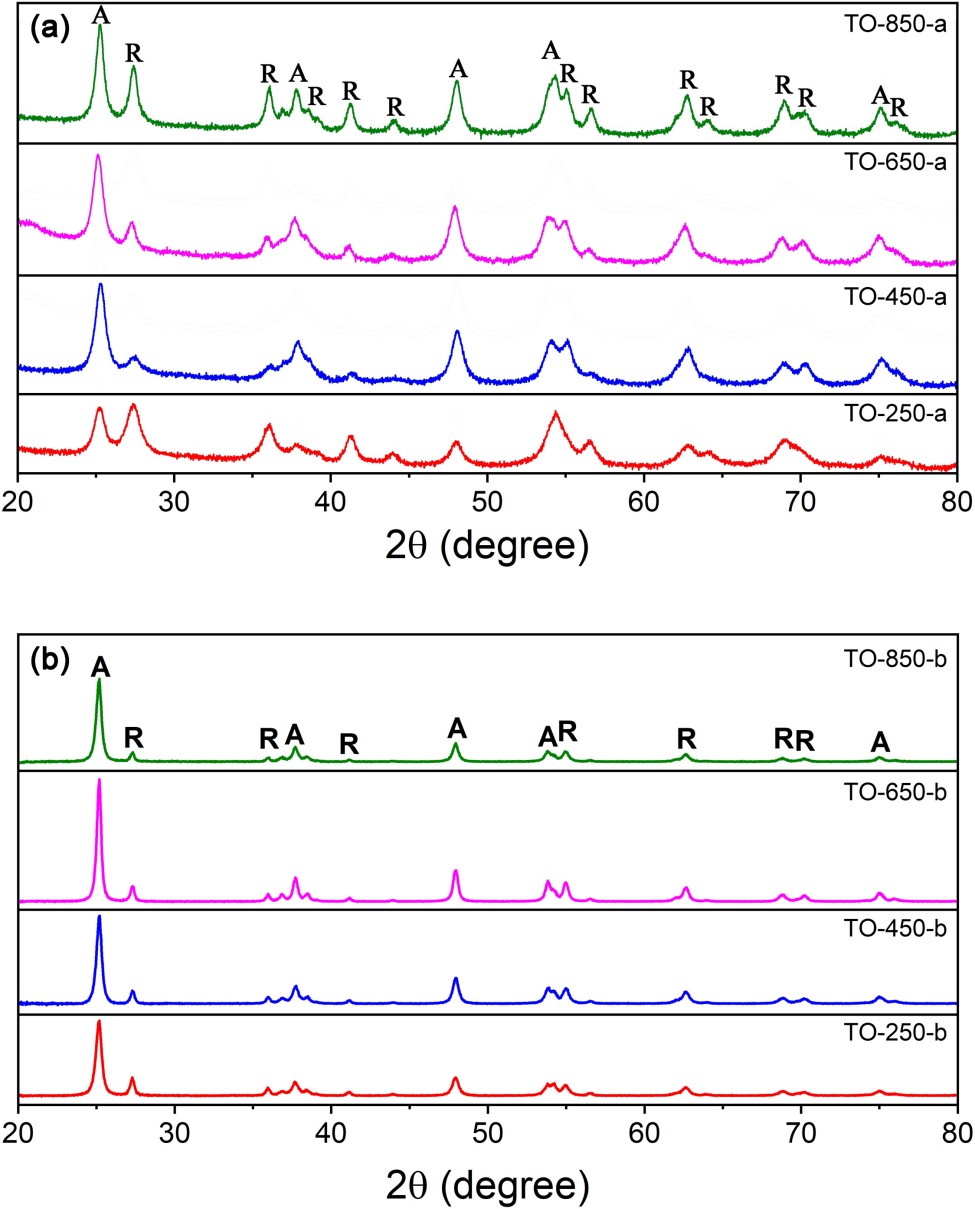
X-ray diffractograms of the TiO_2_ powders: (a) samples of series “a” and (b) samples of series “b” (R: rutile, A: anatase).

Phase composition and crystallites sizes of the TiO_2_ powders were investigated. The X-ray diffractograms of the obtained powders are presented in Figure 1. The calcined TiO_2_ nanopowders show both anatase and rutile phases, corresponding to ICDD database powder diffraction files (PDFs) no. #04-002-2751 and #04-008-7850, respectively.

The ratio between anatase and rutile phases has been calculated using the equations from Spurr and Myers [38], and the medium crystallite diameter has been calculated via the Scherrer equation [39]. Both parameters are presented in Table 2. The anatase crystal structure is dominant for the sample TO-450-a, and this dominance decreases with increasing synthesis pressure. An exception is TO-250-a, that is, the sample synthesized at the lowest pressure and in the presence of the highest C_2_H_4_ sensitizer flow (120 sccm). Here, the rutile crystal structure is the main constituent. The crystallite size increases for both phases from 14 nm and 11 nm to 22 nm for anatase and rutile, respectively, with increasing pressure in the reaction chamber.

**Table 2:** The ratios between anatase and rutile and the crystallite sizes in the powders.

Sample	Anatase [%]	*d*_cryst._ [nm]	Rutile [%]	*d*_cryst._ [nm]

TO-250-a	41.12	14	58.88	11
TO-450-a	90.74	16	9.26	11
TO-650-a	84.21	16	15.79	18
TO-850-a	64.87	22	35.13	22

TO-250-b	77.33	23	22.67	35
TO-450-b	83.30	26	16.70	37
TO-650-b	86.22	33	13.78	40
TO-850-b	87.42	25	12.58	45

**Figure 2:**
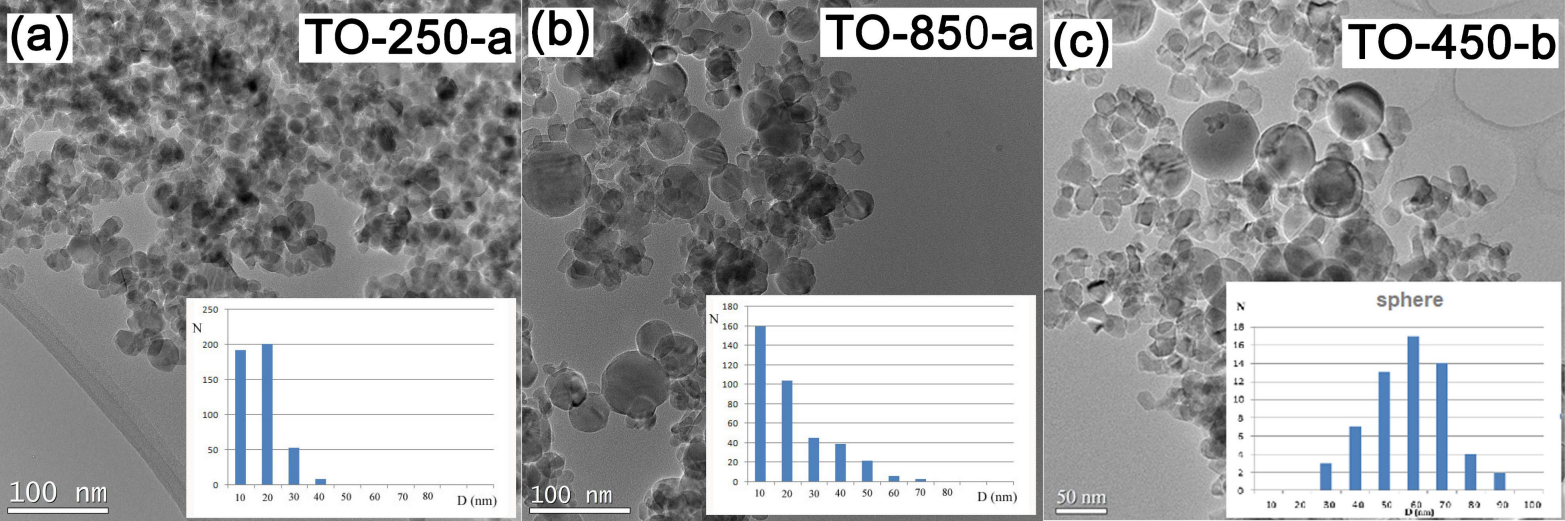
TEM images and the particle distributions: (a) TO-250-a, (b) TO-850-a, and (c) TO-450-b.

The TEM analysis reveals that the TiO_2_ crystallites tend to arrange in bigger spherical particles (Figure 2a,b). This tendency is more pronounced as the pressure in the reaction chamber increases. The average particles sizes for TO-250-a, TO-450-a, TO-650-a, and TO-850-a nanopowders are 17.3, 17.0, 15.5, and 22.0 nm, respectively. These values are in good agreement with the mean crystallites size calculated from XRD investigations.

The number of the bigger particles (60–70 nm) in the TO-850-a powder increases, possibly due to enhanced coalescence in the laser pyrolysis flame at the highest working pressure. Similarly, in the “b” series, the nanoparticles contain crystallites with an average size of 20–25 nm and spherical particles with dimensions between 40 and 80 nm. The biggest spheres of the “b” series were identified in sample TO-450-b (Figure 2c). The highest number of spheres compared to the total number of particles are in sample TO-850-b (7.6%) and the fewest in sample TO-250-b (1.9%).

**Figure 3:**
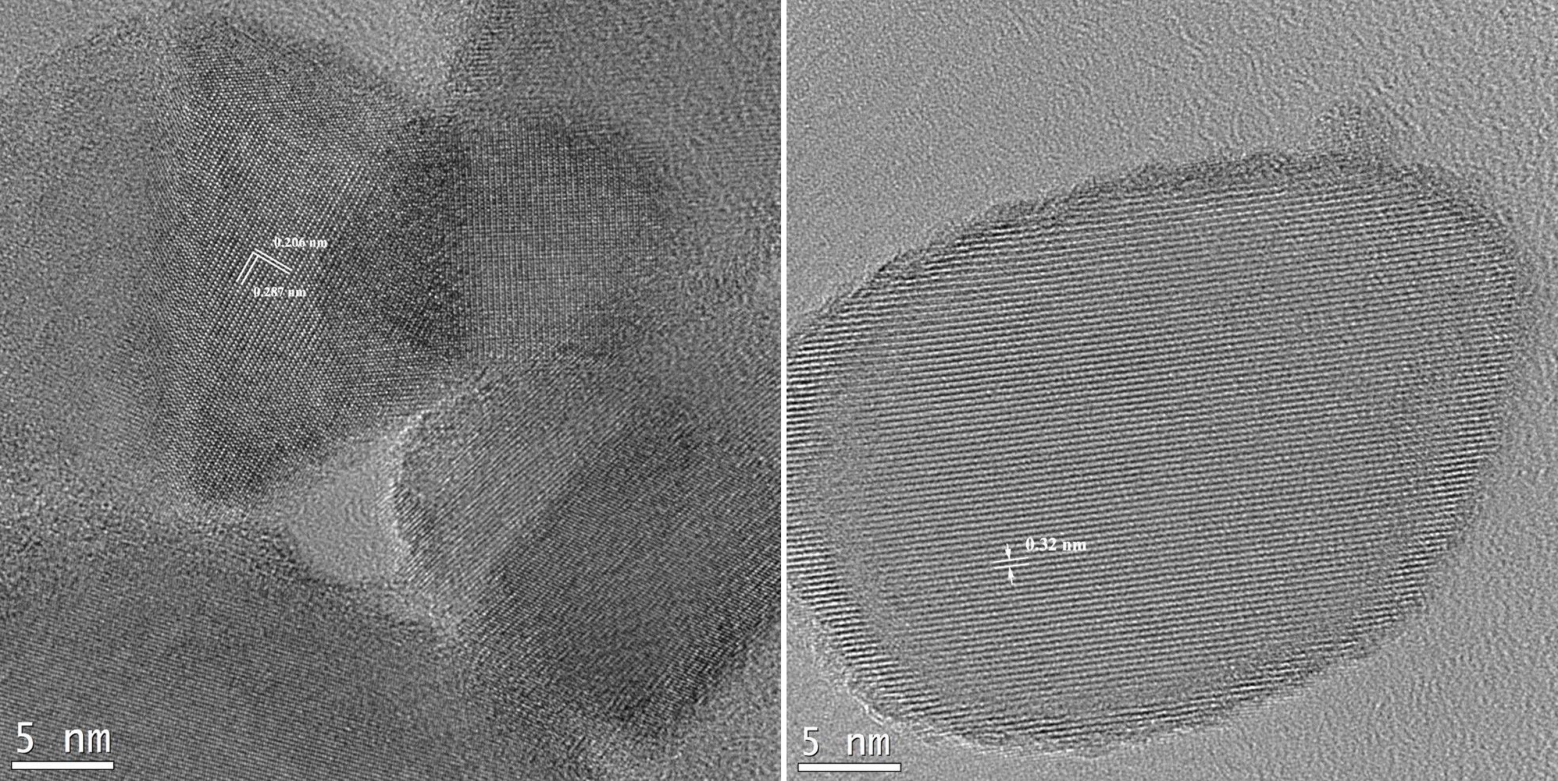
The HRTEM images of the TO-850-a powder.

The HRTEM images (Figure 3) show the crystal structure of the TO-850-a powder with point defects and some residual impurities at the particle surface. The interplanar distance of 0.32 nm (Figure 3, right) corresponds to the *c* axis of the rutile phase (2.96 Å from XRD measurements).

The specific surface areas of series “a” of TiO_2_ powders obtained with increasing pressure in the reaction chamber are 78.0, 82.7, 89.9, and 57.9 m^2^/g, respectively. The correlation between specific surface area and particle/crystallite size is most obvious when comparing the TO-850-a sample with those synthesized at lower pressures. When the average particle size increases, the surface area diminishes.

**Figure 4:**
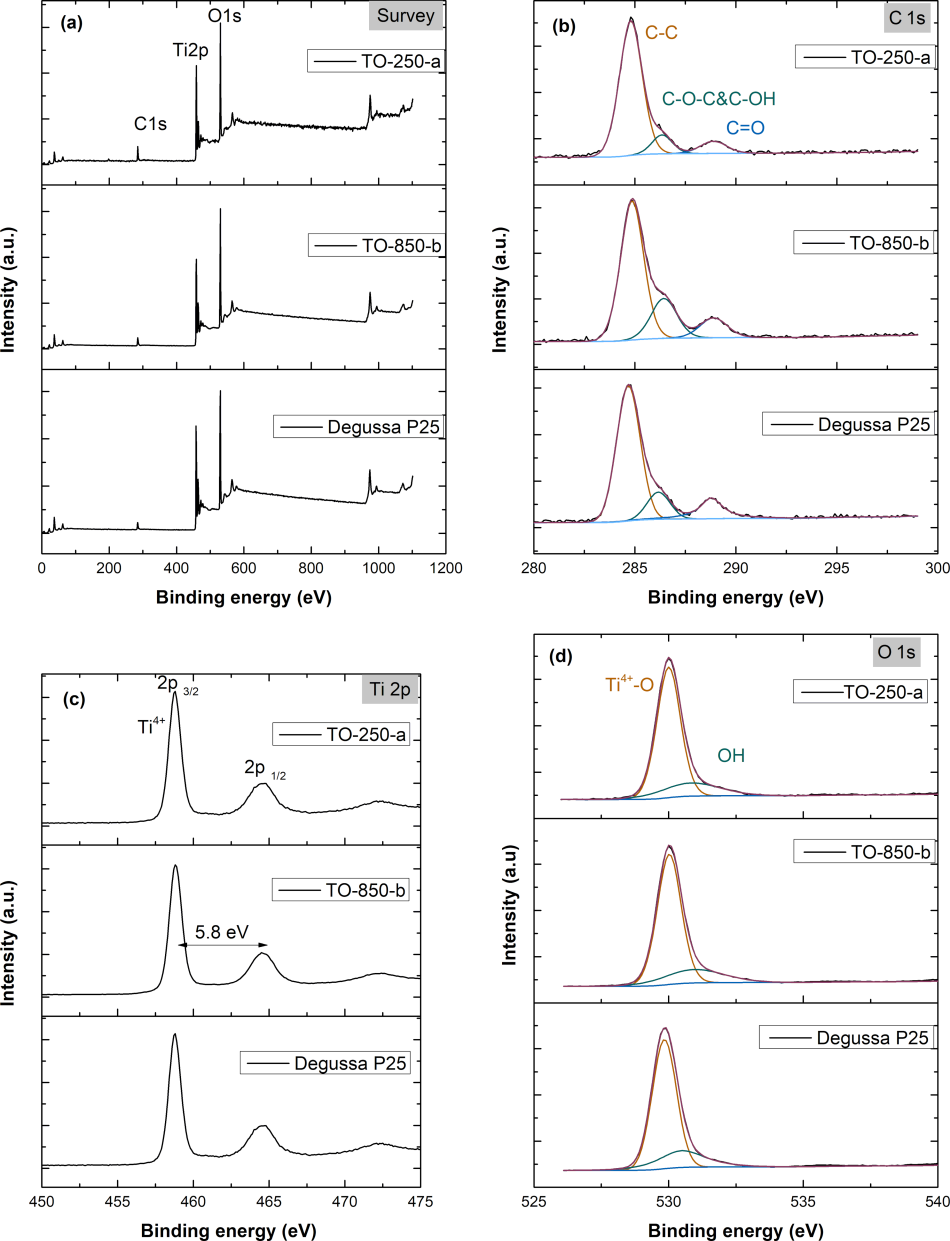
XPS spectra of TO-250-a, TO-850-b, and P25 samples: (a) survey spectra; (b–d) high-resolution XPS spectra of (b) C 1s, (c) Ti 2p, and (d) O 1s core lines.

X-ray photoelectron spectroscopy (XPS) analysis was performed, and the spectral alignment of the binding energy (BE) scale was referenced to adventitious carbon at 284.8 eV [40–41]. Figure 4a shows the full survey scans of TO-250-a, TO-850-b, and commercial TiO_2_ (Degussa P25) samples, indicating the presence of the expected elements Ti, O, and C. The high-resolution XPS spectra of the C 1s, O 1s, and Ti 2p regions of TO-250-a, TO-850-b, and P25 are indicated in Figure 4b–d. The carbon region consists of three singlets with maxima located at 284.8, 286.1, and 289 eV (see Figure 4b). The highest peak located at 284.8 eV originates from the C=C bond, followed by the oxidized carbon forms C–O–C/C–OH (286.1 eV) and O–C–O (289 eV), which probably formed during the synthesis [42]. No C 1s peak at ca. 281 eV (Ti–C bond) was observed, suggesting that carbon does not modify the TiO_2_ phase [43]. Figure 4c shows the Ti 2p peaks, where the Ti^4+^ 2p_3/2_ and Ti^4+^ 2p_1/2_ spin–orbital doublets centered at 459.4 and 464.5 eV correspond to Ti^4+^–O bonds in TiO_2_ [44–47]. The binding energy difference between Ti^4+^ 2p_1/2_ and Ti^4+^ 2p_3/2_ is 5.8 eV, which is similar to the values reported in previous studies [48–49]. The O 1s spectra of pure P25, TO-250-a, and TO-850-b nanoparticles are shown in Figure 4d and were fitted with two peaks. The peaks at binding energies of 529.9 and 530.5 eV are attributed, respectively, to oxygen bound to Ti^4+^ and the adsorption of –OH on the surface [50–51]. The XPS results do not show differences between the two series of samples and do not indicate changes regarding the variation of the nanoparticle surfaces according to the value of the working pressure. Since XPS analysis is a surface measurement and the samples were calcined in air, the possibility to identify the presence of Ti^3+^ species and oxygen vacancies is small, but we do not exclude the possibility that these defects are located on the TiO_2_ surface and that the concentration of defects is below the detection limit of XPS [52–53]. Another explanation would be this: The surface depth sensitivity of XPS is known to be 5–10 nm compared to 1 μm in PL. Hence, this technique provides more details about the species located on the surface and even subsurface [54]. The complementary results regarding our samples are presented below.

One of the most crucial parts in understanding catalytic behavior is the determination of the bandgap value and of shallow donor or acceptor levels, which are localized in the vicinity of the conduction and the valence bands. There are two ways to obtain such knowledge, that is, from optical and luminescence measurements. The former gives us information about electron transitions from the lower to the higher energy levels and the latter about the recombination between free electrons and holes.

**Figure 5:**
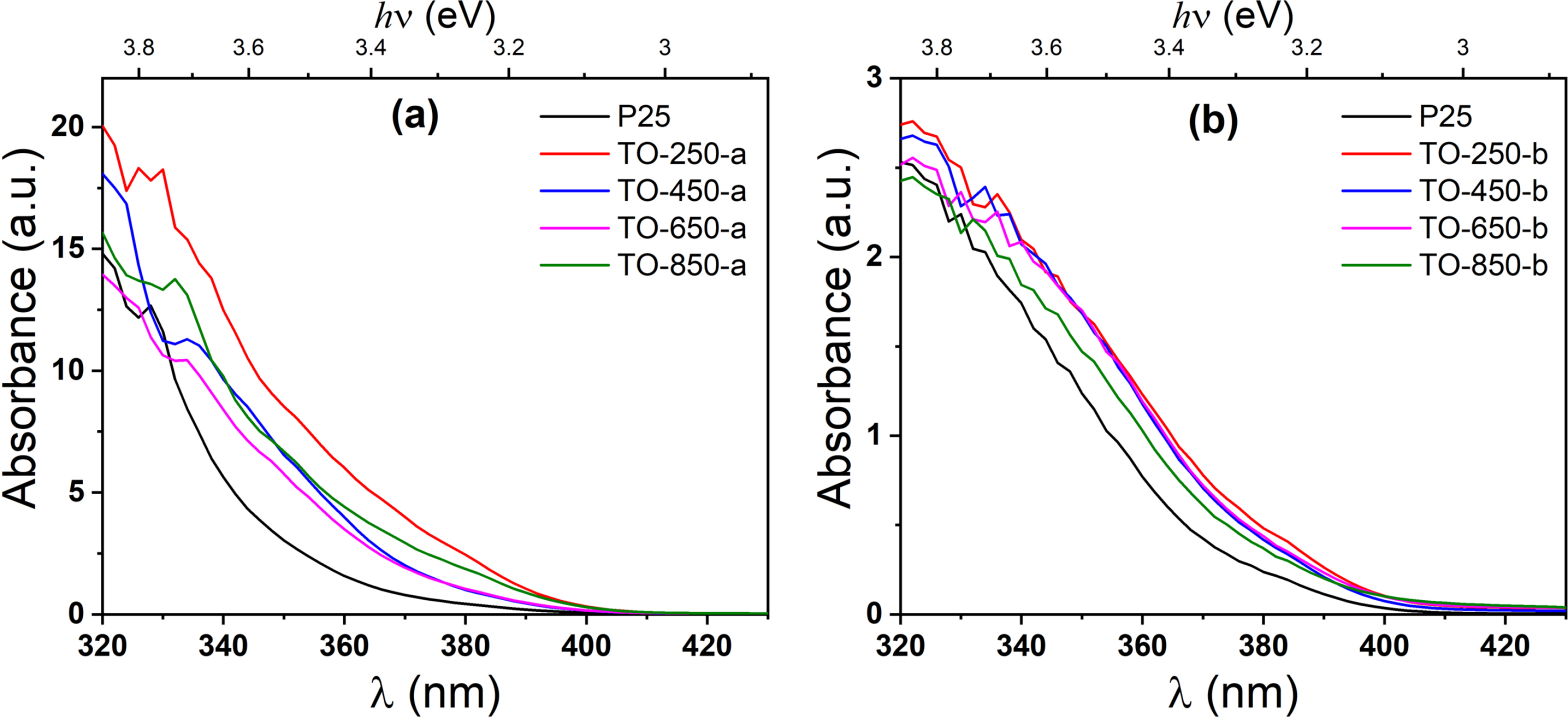
Absorbance of the TiO_2_ samples at room temperature.

The optical properties of the TiO_2_ powders has been investigated by absorbance measurements. Figure 5 presents an example of these measurements of both sample series. In the case of the TiO_2_ material, it is known that it can have two types of transitions, namely direct and indirect transitions, which are related to the crystal structure.

**Figure 6:**
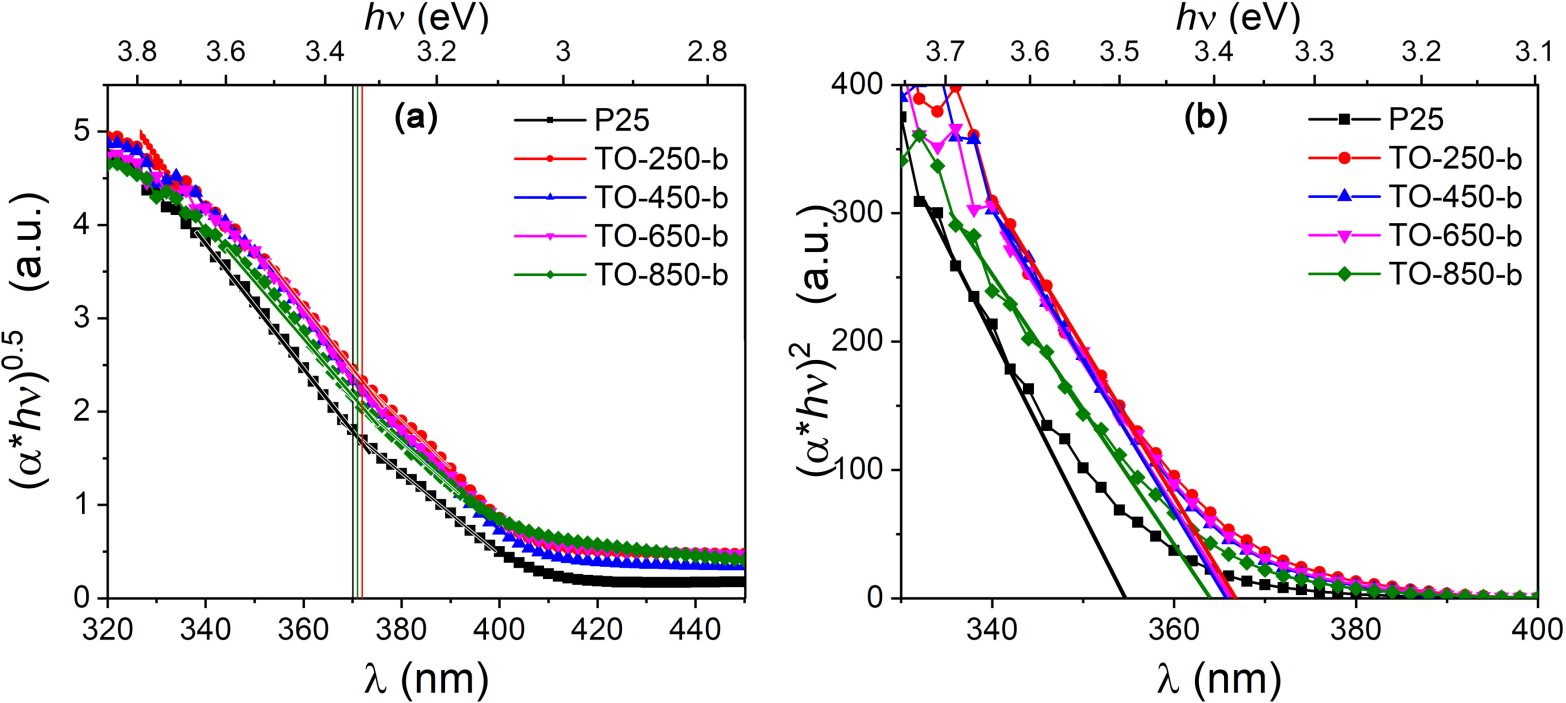
Example of the calculated (a) indirect and (b) direct transitions from sample series “b”.

To distinguish these two types of transition, it is common to use the Tauc plot [55–58], where the absorbance coefficient is multiplied with the photon energy and plotted as an exponential function of the photon energy. The value of the exponent depends on the transition type. For indirect transitions it is 0.5 (Figure 6a), for direct transitions it is 2 (Figure 6b) [55–56]. We presume that in our samples no forbidden transitions occur. The absorbance coefficient was calculated using the following equation [58–59]:


[2] $$\alpha =2.303\frac{\text{absorbance}}{d},$$

where *d* is the thickness of the measured sample.

The bandgap values for bulk anatase and rutile are 3.2 and 2.95 eV, respectively [33,57,60–61]. All calculated bandgap values are listed in Table 3. Our values are slightly higher due to the blueshift with reduction of the size of the powder particles [62–63]. Indirect transitions are more common in both sample groups. The valence bands are the same in P25 and our TiO_2_ powders, indirect transitions have almost the same values for all measured samples, and the differences among the values could be due to calculus errors. In the case of direct transitions, the values obtained in our powders are smaller. A possible explanation is the smaller volume of the particles with anatase crystal structure or the formation of shallow donor levels near the conduction band [64–65].

**Table 3:** Calculated bandgap values.

	Indirect		Direct	
	
Sample	Series “a” [eV]	Series “b” [eV]	Series “a” [eV]	Series “b” [eV]

P25	3.03	3.35	3.64	3.49
TO-250	3.00	3.33	3.49	3.37
TO-450	3.07	3.37	3.50	3.38
TO-650	3.05	3.32	3.52	3.37
TO-850	3.00	3.34	3.50	3.40

**Figure 7:**
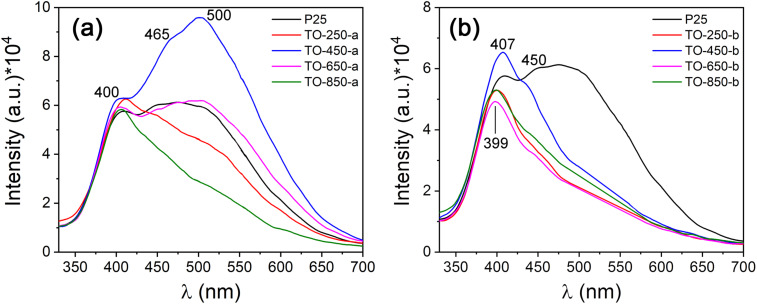
Photoluminescence of the TiO_2_ powders under λ_exc_ = 260 nm at room temperature: (a) samples of series “a” and (b) samples of series “b”.

Photoluminescence (PL) spectra at 260 nm excitation wavelength are presented in Figure 7. The most important band is the band located at 400 nm (3.10 eV), but there are some other bands at 450 nm (2.75 eV), 465 nm (2.66 eV), and 500 nm (2.48 eV). The band at 400 nm is complex, and its maximum shifts depending on the intensity of each component. For example, the maximum of the 400 nm band of the TO-250-a and TO-450-b samples are shifted to lower energies, which is caused by the more intense PL component in the region of longer wavelengths. Other PL bands represent the recombination probability at energy levels located more deeply in the forbidden bandgap. Some of them could be donor levels, for example chlorine atoms [66], but most probably they are acceptor levels created by titanium vacancies [67–68].

Band-to-band transitions have not been detected as separate PL bands, taking into account the obtained bandgap values from the optical properties. But, they are surely a part of the complex PL band at 400 nm, the resolution of which into the components requires another investigation. Oxygen and titanium vacancies have been found. These points defects are created most probably at the surface of the powder particles because in the HRTEM figures we do not see any dislocation or other crystal structure modification inside the particles. Another explanation is that photons emitted from the depth of the particles are reabsorbed in the superficial zone and are then reemitted. There is also a high probability that free electrons are trapped by donor levels (*V*_O_) [65,67] and that recombination processes are taking place at that level. This also applies to free holes trapped at acceptor levels [68], which cannot escape as easily as electrons from the donor level.

### Generation of hydroxyl radicals

Reactive oxygen species are usually involved in the photodegradation of organic compounds. For example, the hydroxyl radical (•OH) is a strong oxidizer. The generation of (•OH) over the samples under simulated solar light irradiation (AM 1.5) has been evaluated according to the PL emission from 451 nm, attributable to the presence of umbelliferone, a derivative of coumarin resulting from the interaction with photogenerated hydroxyl radicals.

Despite the fact that radical trapping was performed in aqueous solution, it can be indicative for the ability of the catalyst surface to generate hydroxyl radicals in the present investigated system. The main reactions leading to (•OH) formation are the following:


[3]

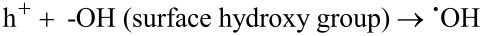



or


[4]

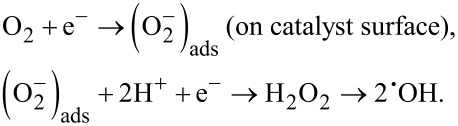



**Figure 8:**
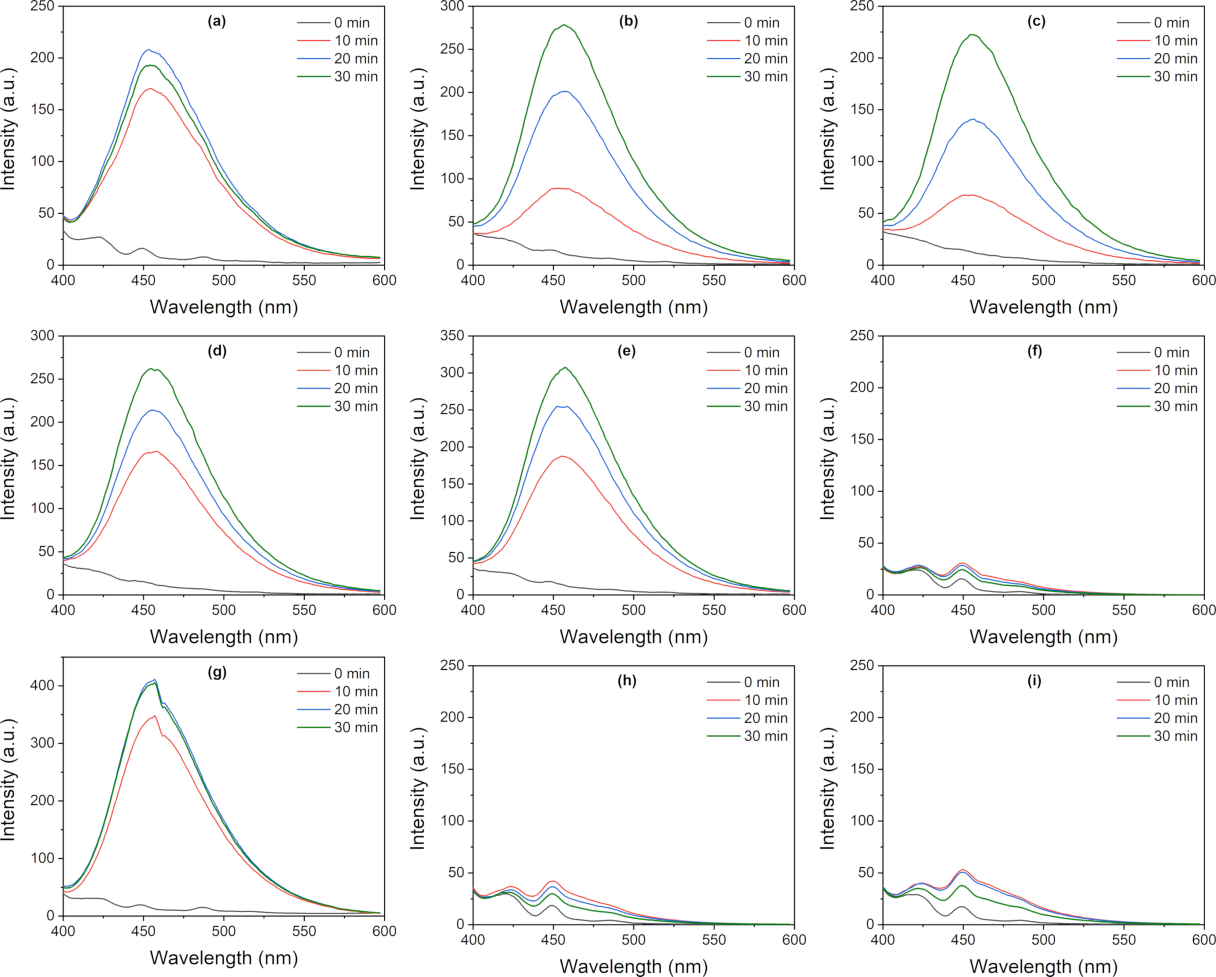
Generation of hydroxyl radicals under simulated solar irradiation over the investigated catalysts as a function of the time. The emission at 451 nm is characteristic to the presence of umbelliferone: (a) P25, (b) TO-250-a, (c) TO-450-a, (d) TO-650-a, (e) TO-850-a, (f) TO-250-b, (g) TO-450-b, (h) TO-650-b, and (i) TO-850-b.

Figure 8 shows a significant ability of TO-250-a, TO-450-a, TO-650-a, TO-850-a, and P 25 catalysts to generate •OH, indicating a presumable activity for CO_2_ generation. In contrast, the samples of series “b” are almost inactive. Only TO-450-b can produce hydroxyl radicals.

### Photocatalytic performance of the nanopowders

These experiments have been conducted in environments with low oxygen concentration. Ethanol vapors play a double role here, that is, they generate hydrogen by photodehydrogenation and also undergo oxidation to carbon dioxide and water under the action of simulated sunlight in the presence of oxygen. The main intermediate product in the process of hydrogen photogeneration is acetaldehyde as intermediate, which can be further oxidized to CO_2_ and H_2_O, according to the following chemical equations:


[5] $${\text{C}}_{2}{\text{H}}_{5}\text{OH}\to {\text{CH}}_{3}\text{CHO}+{\text{H}}_{2},$$

[6] $${\text{CH}}_{3}\text{CHO}+2.5{\text{O}}_{2}\to 2{\text{CO}}_{2}+{\text{H}}_{2}\text{O.}$$

### Hydrogen generation

**Figure 9:**
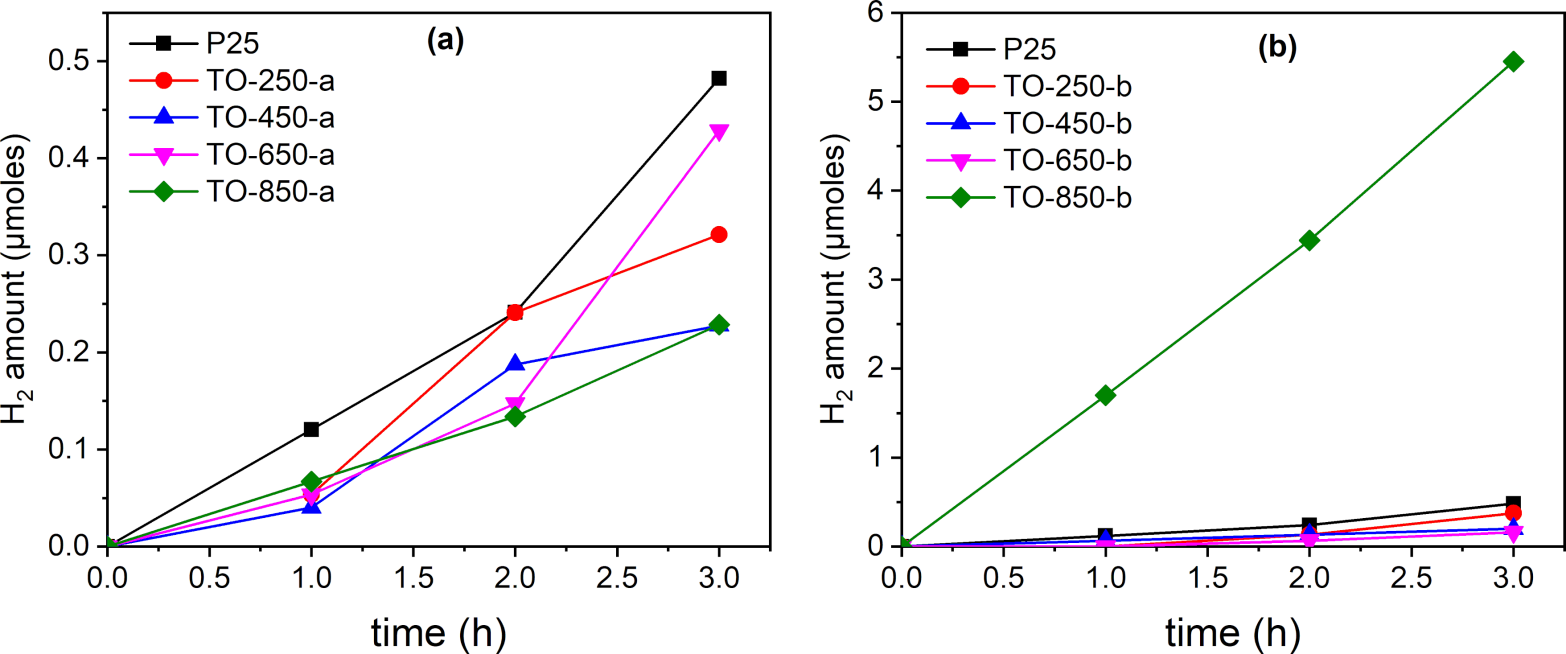
Photocatalytic H_2_ evolution from C_2_H_5_OH vapors over both catalyst series under simulated solar light irradiation: (a) series “a” and (b) series “b”.

As can be seen in Figure 9, the H_2_ generation performance of the tested titania nanoparticles greatly differs. The TO-850-b sample exhibits the highest activity for H_2_ photogeneration, reaching 5 µmol H_2_ after 3 h of reaction. The entire sequence of TO-250-b, TO-450-b, and TO-650-b catalysts proves to have a higher activity for H_2_ generation than TO-250-a, TO-450-a, and TO-650-a, which are quite close to P 25 in terms of an almost insignificant H_2_ production. This clear difference between the two catalysts series can be related to structural characteristics providing different densities of photogenerated charges (electrons) to react with protons available at the surface.

### Carbon dioxide evolution

**Figure 10:**
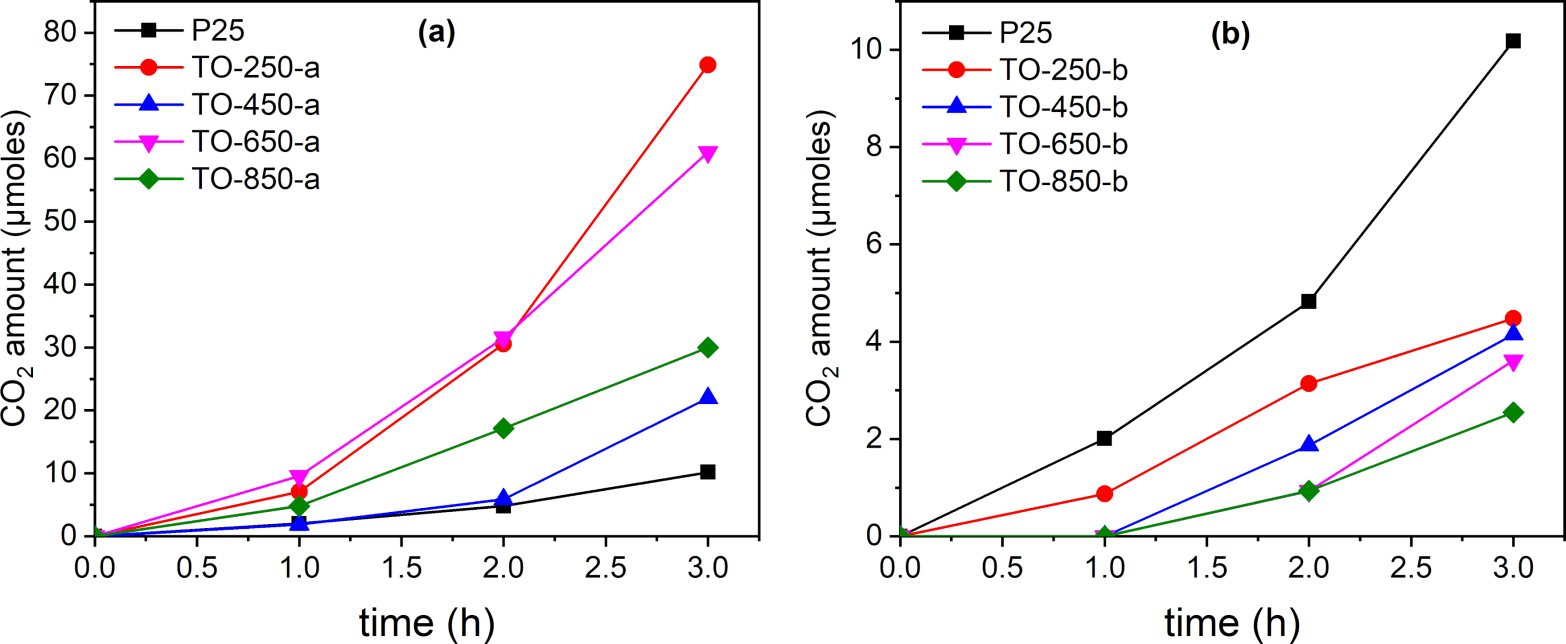
Photocatalytic CO_2_ evolution from C_2_H_5_OH vapor over TiO_2_ under simulated solar light irradiation: (a) series “a” and (b) series “b”.

The photo-oxidative conversion of ethanol to CO_2_ (and water) under simulated sunlight irradiation is depicted in Figure 10, revealing the different kinds of behavior of the sample series “a” and “b”. All samples of series “a” show a significant activity regarding the mineralization of ethanol in the gas phase. The highest activity was measured for TO-250-a, which generated around 70 µmol CO_2_ after 3 h. All catalysts of this series exhibit a higher activity than P25. Also, an increase of the CO_2_ formation rate after the first hour of irradiation can be observed, probably due to the mineralization of the previously generated intermediates. The catalysts of the series “b” are less active than P 25 regarding the mineralization to CO_2_. A very good correlation can be established between these results and Figure 8 illustrating the hydroxyl radical formation. It is obvious that all catalysts of the first series generate high amounts of hydroxyl radicals and, consequently, trigger ethanol photomineralization. It should be noted that the TO-450-b sample also produced hydroxyl radicals and shows a straight increase of CO_2_ formation after 1 h of irradiation. Probably, a longer irradiation time would be beneficial for ethanol photomineralization over this sample. Also, TO-650-b shows a linear increase of activity after 2 h of irradiation.

**Figure 11:**
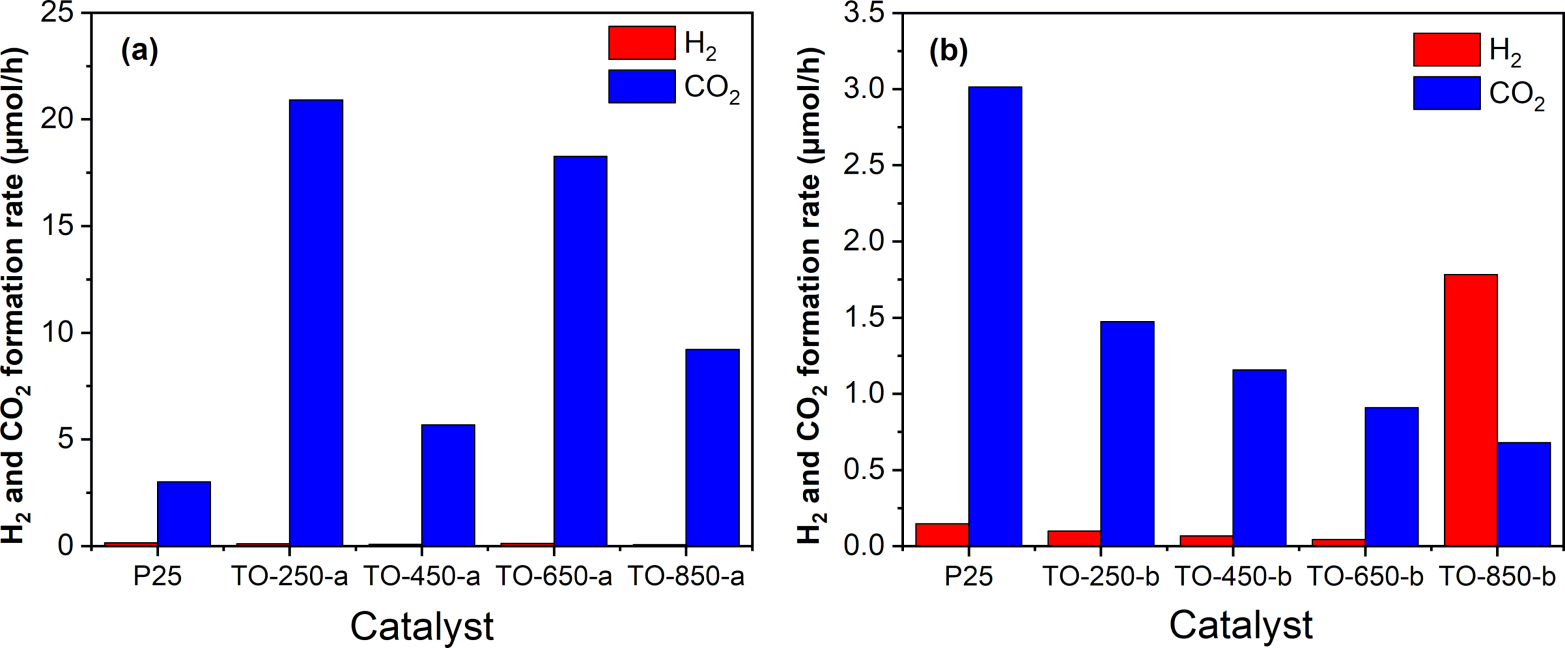
Comparative H_2_/CO_2_ formation rate over titania nanopowders in ethanol vapor environments: (a) series “a” and (b) series “b”.

Figure 11 is illustrative for the different kinds of behavior of the two catalyst series regarding ethanol photodegradation. For the ethanol photodegradation carried out over the TO-250-a, TO-450-a, TO-650-a, and TO-850-a catalysts, the formation rate of CO_2_ is significantly higher than the formation rate of H_2_. This indicates a potential use for depollution since TiO_2_ is an environmentally friendly, non-toxic, and inexpensive material. More than that, these tests used solar light, which is a regenerable energy source. The investigated TiO_2_-based catalysts lead to ethanol photomineralization under simulated solar light, indicating an efficient use of UV light from the solar spectrum, but also the presence of optically and catalytically active defects in the structure of the catalysts.

Figure 11 shows also a lower CO_2_ formation rate for the second catalyst series with a simultaneously increasing H_2_ formation rate, especially for TO-850-b. From this perspective, it might be interesting to consider these catalysts for use and further modification regarding H_2_ production. Table 4 shows an increase of acetaldehyde formation for the catalyst series “b” relative to series “a”, especially for TO-850-b. This observation is in line with the highest hydrogen production obtained by using this catalyst.

**Table 4:** Comparative formation of acetaldehyde after 180 min of irradiation over the investigated catalysts.

Time reaction (min)	Amount of CH_3_CHO [µmol]								

	P25	TO-250-a	TO-450-a	TO-650-a	TO-850-a	TO-250-b	TO-450-b	TO-650-b	TO-850-b

0	0	0	0	0	0	0	0	0	0
30	36.29	14.30	4.74	4.65	8.52	50.20	23.00	50.00	50.30
60	65.11	31.42	20.00	11.67	18.00	84.50	41.90	60.00	87.20
90	76.72	42.11	26.00	16.80	26.19	90.40	53.40	63.00	106.00
120	87.19	52.40	39.87	21.80	38.00	92.40	56.00	71.00	111.00
150	92.20	62.41	40.46	27.00	50.00	97.00	59.00	74.00	113.00
180	97.67	71.07	42.00	36.80	55.00	90.00	63.00	80.00	114.00

## Conclusion

This study describes the photocatalytic degradation of ethanol vapors under simulated solar light and low oxygen concentration using TiO_2_ nanoparticles obtained by laser pyrolysis. The final products are CO_2_, H_2_O, and H_2_. The average particle sizes are between 15 and 22 nm with anatase being the predominant crystalline phase and rutile being a minor fraction. An exception to this is the sample synthesized at the lowest pressure without supplementary air flow. Two series of samples were tested in ethanol photodegradation experiments, observing that all samples from series “a” have a higher photocatalytic activity towards the oxidation of ethanol to CO_2_ than the reference sample Degussa P25, especially the small nanoparticles obtained at the lowest pressure (250 mbar). In contrast, the larger nanoparticles obtained in the “b” series at the highest pressure (850 mbar) contribute to a slight improvement regarding the production of H_2_, compared to the commercial reference sample. The ethanol photodegradation under simulated solar light leads to valuable results concerning different TiO_2_ functional properties depending on the synthesis conditions. Also, a potential application both for the degradation of organic compounds and the production of hydrogen has been revealed.

## Materials and Methods

**Figure 12:**
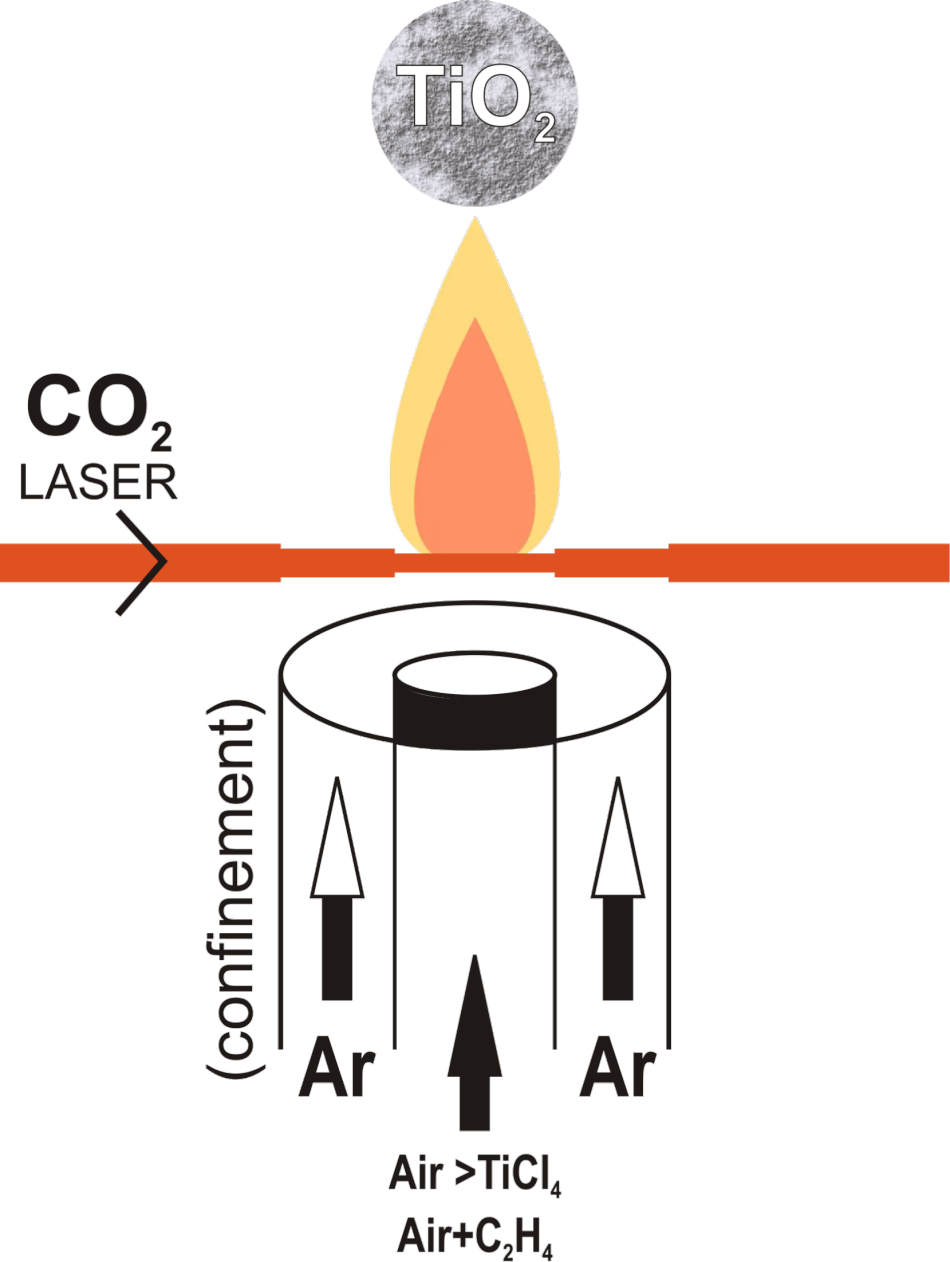
Schematic representation of the laser pyrolysis method.

Laser pyrolysis was used to obtain powder of TiO_2_ in a similar manner as described in our previous studies [69–70]. The laser radiation was generated by a continuous CO_2_ laser with 10.55 μm wavelength and maximum power of 450 W. Ethylene was used to absorb the infrared laser radiation and transfer the energy to the precursor molecules, thus playing the role of a sensitizer. The reaction took place in the volume delimited by the orthogonally intersection of the laser beam with the precursor flow (Figure 12).

The precursors were injected through the central nozzle. The reactive flow was a mixture of synthetic air (Siad 99.99% purity) as oxidizer, C_2_H_4_ (Siad 99.5% purity) as sensitizer, and TiCl_4_ vapor (Aldrich 98% purity) as Ti precursor. Synthetic air was used as carrier of gaseous TiCl_4_ from a liquid reservoir (via a bubbler) and as oxidizer (Table 5). The co-axial flow of Ar (Siad 99.98% purity) confined the inner reactive flow after passing through an external annular inlet. The powder collection time was ca. 1 h. The first powders (denominated as series “a”) were further calcined at 450 °C for 5 h in order to minimize the traces of carbon from ethylene decomposition and of chlorine from TiCl_4_. Because the flow of entrained precursor vapors diminished with increasing pressure of the carrier gas, in the first series of experiments we increased the carrier gas flow at higher pressures to not reduce the TiCl_4_ flow too much. Also, a supplementary air flow was used in these experiments, with the exception of those at the lowest pressure. Also, in this first series of experiments, we gradually decreased the C_2_H_4_ sensitizer flow with increasing pressure in order to maintain a stable burning flame. The process conditions were further modified (by enhancing the air carrier flow and diminishing the C_2_H_4_ flow and employing the same carrier and supplementary air and ethylene flows at all pressures) in order to obtain higher-purity TiO_2_ nanoparticles that do not require post-synthesis treatment. These samples were grouped in the second batch of samples, that is, in series “b”.

**Table 5:** Process parameters of laser pyrolysis synthesis of titania nanoparticles.

Sample	Φ_Ar(conf.)_ [sccm]	Φ_air_ [sccm]	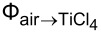 [sccm]	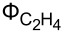 [sccm]	*P* [mBar]	Laser power [W]

TO-250-a	1800	—	80	120	250	450
TO-450-a	1800	100	120	100	450	450
TO-650-a	1800	200	160	80	650	450
TO-850-a	2000	200	200	60	850	450

TO-250-b	2000	200	300	50	250	450
TO-450-b	2000	200	300	50	450	450
TO-650-b	2000	200	300	50	650	450
TO-850-b	2000	200	300	50	850	450

The elemental composition of the TiO_2_ powders was estimated by EDS performed inside a scanning electron microscope, FEI Quanta Inspect S, at 15 kV in high vacuum. The crystalline structures and phase concentrations were determined from X-ray diffraction (XRD) patterns, measured by an X-ray diffractometer Panalytical X’Pert MPD theta–theta, and the morphological properties were determined by transmission electron microscopy (TEM), high-resolution transmission electron microscopy (HRTEM), and selected-area electron diffraction (SAED) measurements using a JEM ARM 200F analytical microscope (Jeol, Japan). The specific surface area was measured using a BET flowing gas surface area analyzer, Horiba SA-9600, with a 30% N_2_/70% He gas mixture. Photoluminescence measurements were carried out using a Horiba Flourolog-3. The excitation source was a xenon lamp at λ_exc_ = 260 nm. All samples have been irradiated at room temperature and under the same conditions. Diffuse reflectance UV–vis measurements were performed by using a spectrophotometer Perkin Elmer Lambda 35 with an integrating sphere in the 200–1100 nm range. The measured reflectance data were converted to absorption data using the Kubelka–Munk function *F*(*R*).

For the XPS analyses we employed an ESCALAB Xi+ (Thermo SCIENTIFIC Surface Analysis) apparatus with an Al Kα radiation source (*h*ν = 1486.2 eV) using the C 1s level (284.4 eV) as the energy reference. The superficial chemical compositions as well as the oxidation states were found from the XPS spectra by using the “Avantage” software, version 5.978.

### ROS identification

Trapping of •OH radicals was performed with 10 mM coumarin (Merck) solution and 0.001 g suspended catalyst exposed to simulated solar irradiation. The formation of a fluorescent compound, namely umbelliferone, was monitored with a Carry Eclipse fluorescence spectrometer, slits set to 10 nm in excitation and emission, λ_exc_ = 330 nm.

The experimental procedure for photocatalytic tests started with dispersing a uniform layer of 0.01 g of titania photocatalyst nanopowder on an area of about 3.6 cm^2^. This photoactive surface was subsequently exposed to simulated sunlight. Ethanol (7.2 µL) was injected into the photoreactor with a volume of about 120 cm^3^ containing 0.3% O_2_ in Ar. The temperature inside the photoreactor was maintained constant at 18 °C with a cryostat. The AM 1.5 solar light (1000 W/m^2^) was provided by a Peccell L01 solar simulator. For each test, 200 µL gas samples were taken from the photoreactor every 30 min and analyzed with two gas chromatographs equipped with either a flame ionization detector (FID, Agilent 7890A) or a thermal conductivity detector (TCD, Buck Scientific, model 910). The total time of a photocatalytic test was 180 min. The photoreactor works thus under static conditions, which differs from the dynamic conditions used by other researchers where a continuous flow of ethanol vapors (mixed with water vapors) was employed via bubbling [18,71].
